# The mental health benefits of community helping during crisis: Coordinated helping, community identification and sense of unity during the COVID‐19 pandemic

**DOI:** 10.1002/casp.2520

**Published:** 2021-04-05

**Authors:** Mhairi Bowe, Juliet R. H. Wakefield, Blerina Kellezi, Clifford Stevenson, Niamh McNamara, Bethany A. Jones, Alex Sumich, Nadja Heym

**Affiliations:** ^1^ Department of Psychology Nottingham Trent University Nottingham UK

**Keywords:** community, COVID‐19, helping, mental health, social identity, volunteering, well‐being

## Abstract

Communities are vital sources of support during crisis, providing collective contexts for shared identity and solidarity that predict supportive, prosocial responses. The COVID‐19 pandemic has presented a global health crisis capable of exerting a heavy toll on the mental health of community members while inducing unwelcome levels of social disconnection. Simultaneously, lockdown restrictions have forced vulnerable community members to depend upon the support of fellow residents. Fortunately, voluntary helping can be beneficial to the well‐being of the helper as well as the recipient, offering beneficial collective solutions. Using insights from social identity approaches to volunteering and disaster responses, this study explored whether the opportunity to engage in helping fellow community members may be both unifying and beneficial for those engaging in coordinated community helping. Survey data collected in the UK during June 2020 showed that coordinated community helping predicted the psychological bonding of community members by building a sense of community identification and unity during the pandemic, which predicted increased well‐being and reduced depression and anxiety. Implications for the promotion and support of voluntary helping initiatives in the context of longer‐term responses to the COVID‐19 pandemic are provided. Please refer to the Supplementary Material section to find this article's Community and Social Impact Statement.

## 1 INTRODUCTION

COVID‐19 constitutes an unparalleled “social threat” (Banerjee & Rai, 2020). Global government restrictions to limit viral spread have disrupted communities and exacerbated loneliness, especially among the socially, economically and medically vulnerable (UN, 2020) whilst also restricting access to vital community‐based social support. Research that helps identify methods of protecting community members' mental health and well‐being whilst maintaining social cohesion is thus imperative (O'Connor et al., 2020; Van Bavel et al., 2020). However, whilst the pandemic has created an urgent need for people to find opportunities for social connection to support their mental health, it has also created demand for community members to volunteer time to support others. We contend that these parallel processes may themselves offer a solution.

Despite the potentially corrosive effects of the pandemic on community life, communities have typically responded by aiding their most vulnerable members giving rise to a global proliferation of community‐based mutual‐aid groups and an influx of volunteers to formal roles (Drury & Tekin Guven, 2020; Monbiot, 2020; Tiratelli & Kaye, 2020). This response has provided essential assistance and supplemented governmental attempts to support citizens. It has been suggested that capitalizing on these offers of help benefits the community at the expense of volunteers and concerns remain about the long‐term well‐being‐related costs of volunteering (Gilbert, 2020). However, this neglects the inherently social and beneficial dimensions of helping. For example, the upsurge in collective helping accords with emerging understanding of the benefits of volunteering for building community identity and support, which in turn enhances well‐being (Bowe et al., 2020; Gray & Stevenson, 2020). It also accords with research pointing to the pivotal role of strong social relationships and collective support provision in effective long‐term responses to emergencies (Drury, Brown, González, & Miranda, 2016).

In this article, we provide an overview of the negative impacts of the pandemic on the mental health of local communities and the observed community responses. We outline a key theoretical framework (the Social Identity Approach to Health; C. Haslam, Jetten, Cruwys, Dingle, & Haslam, 2018), which has demonstrated the health benefits of community identification and resilience in disasters, then detail more recent work on the effects of volunteering on local community identity and well‐being. Inspired by this theoretical framework, we report novel work exploring the relationships between help‐giving, community relationships and unity during the pandemic and illustrate the pathways through which help‐giving may predict residents' mental health and well‐being.

### 1.1 COVID‐19's impact on community mental health/well‐being

The COVID‐19 pandemic has significant implications for mental health and well‐being, to the extent that these outcomes have been labelled a “second pandemic” (Choi, Heilemann, Fauer, & Mead, 2020). The social and economic consequences of COVID‐19 constitute a stressor capable of causing chronic anxiety (Holmes et al., 2020), particularly in communities where death rates are high (Lima et al., 2020), and for populations with pre‐existing health issues (Brooks et al., 2020). Recent data link fear of COVID‐19 with both health‐related and generalized anxiety (Lee, Jobe, Mathis, & Gibbons, 2020), but its impact may also be more nuanced. For instance, widespread financial insecurity and established relationships between financial stress and mental health (Frasquilho, de Matos, Gaspar, & de Almeida, 2016) suggest the pandemic's economic consequences will also impact well‐being (Brooks et al., 2020). Other stressors such as home‐schooling and increases in domestic violence are also likely to elevate mental ill‐health (Social Care Institute for Excellence, 2020). The impact of COVID‐19 on depression has already been demonstrated in China, especially for those with mental health issues (Wang et al., 2020). However, the pandemic is also likely to affect mental health for those without pre‐existing conditions. Fear of COVID‐19 has been found to predict depression beyond the impact of pre‐existing vulnerabilities and socio‐demographics factors (Lee et al., 2020), and those who come in to contact with COVID‐19 are also at risk of pandemic‐related vicarious traumatization (Z. Li et al., 2020).

The impact of the pandemic on social life is likely to exacerbate these effects. Limited access to community activities, changes in living circumstances and social isolation are likely to be detrimental to mental health (O'Connor et al., 2020; Thompson, Garfin, Holman, & Silver, 2017). Stress appraisal and emotion regulation are also likely to be weakened due to reduced access to fellow social group members (C. Haslam et al., 2018; Williams, Morelli, Ong, & Zaki, 2018), whilst distancing and reduced contact encourages loneliness and its mental health consequences (Cacioppo, Hughes, Waite, Hawkley, & Thisted, 2006; Holmes et al., 2020). Around one third of UK survey respondents report experiencing negative emotional consequences of pandemic‐related disconnection, and this is particularly anxiety‐inducing for those with challenging living circumstances, lack of support networks and existing conditions (L. Z. Li & Wang, 2020). A series of studies exploring “corona‐related loneliness” highlight the need for psychologically meaningful sources of social connection and suggest this should be central to interventions aimed at protecting mental health (Hoffart, Johnson, & Ebrahimi, 2020; Horesh, Kapel Lev‐Ari, & Hasson‐Ohayon, 2020, particularly as typical sources of formal mental health support may be less accessible (O'Connor et al., 2020). For these reasons, identifying alternative routes to promoting community health and well‐being must also be prioritized (Holmes et al., 2020).

### 1.2 Community helping as a pathway to mental health/well‐being

Among those most affected by the pandemic lockdown were older and medically vulnerable adults who required urgent assistance to meet their needs for food and medical supplies. In the pandemic's early stages, these needs often went unmet by local services. In most areas across the UK, residents associations, often under the umbrella of “COVID‐19 Mutual‐Aid Groups”, stepped in to provide practical and emotional support to vulnerable residents (Booth, 2020; Stansfield, Mapplethorpe, & South, 2020) and engagement with mutual aid groups involved both existing volunteers and community members who had never volunteered before (Tiratelli & Kaye, 2020). Engagement in the UK exemplified a global upsurge in volunteering activity, whereby individuals engaged in unprecedented levels of locale‐based organized helping activities via community‐based mutual aid groups (Monbiot, 2020).

The role of communities in providing social and psychological resources to residents is well‐established in social psychology. The Social Identity Approach to Health demonstrates that a range of physical and mental health outcomes result from identification with social groups, constituting a “social cure” (C. Haslam et al., 2018). Social relationships characterized by belonging, trust and support are therefore likely to be vital resources in the pandemic response (Jetten, Reicher, Haslam, & Cruwys, 2020). Cohesive residential community groups are known to be a particularly important source of identity and belonging, valuable for the generation and sharing of support and for residents' health and well‐being (Ehsan, Klaas, Bastianen, & Spini, 2019; Fong, Cruwys, Haslam, & Haslam, 2019; McNamara, Stevenson, & Muldoon, 2013). Meanwhile, neighbour proximity affords opportunities for shared interests, common fate and interactions, thus potentially resulting in meaningful social bonds (Easterbrook & Vignoles, 2015). Neighbourhoods also provide collective resilience to shared challenge. In areas of deprivation, community identification has been shown to predict residents' well‐being via feelings of collective efficacy (McNamara et al., 2013). Whilst, neighbourhood identification has also been linked with increased support, well‐being and resilience during regeneration (Heath, Rabinovich, & Barreto, 2017) and moderates the negative relationship between low socioeconomic status and residents' health (Fong et al., 2019). In sum, during the mundane interactions of everyday life, residential community identification serves to enhance and protect residents' mental health and well‐being.

These community relationships are also critical during disasters because they motivate cohesive, prosocial responses and coalesce around a sense of solidarity and support (Drury et al., 2016). In effect, the shared identity that emerges from perceptions of common fate during disasters leads to an enhanced and beneficial experience of unity among communities thus allowing for the development of collective efficacy during times of stress (Drury, Cocking, & Reicher, 2009; Ntontis, Drury, Amlôt, Rubin, & Williams, 2018). The COVID‐19 pandemic has aroused these feelings of shared fate and solidarity, expressed in the form of community‐based support and coordinated action (Drury & Tekin Guven, 2020). As well as feelings of unity and solidarity, previous research has found community‐based responses to disaster can protect the mental health of those involved, buffering them from trauma (Muldoon et al., 2017). Importantly, this process has also been linked with spontaneous engagement in acts of helping and solidarity during chronic stress and disaster (Alfadhli, Cakal, & Drury, 2019; Vezzali, Drury, Versari, & Cadamuro, 2016). The decisive pro‐social responses needed to bolster support delivery for the vulnerable are therefore vital for effective pandemic management (Drury, Reicher, & Stott, 2020; Elcheroth & Drury, 2020) but may also constitute a psychological resource to protect community members' mental health and well‐being. Clearly these explanatory frameworks promise much in the way of interpreting and supporting effective inclusive community responses to COVID‐19 (Templeton et al., 2020); however, the precise pathways linking voluntary helping within community settings to potential health/well‐being outcomes during crisis are currently undetermined.

### 1.3 Volunteering and community identity

A range of evidence suggests that helping behaviours have a positive impact on health and well‐being. Longitudinal and cross‐cultural studies show volunteering results in increased happiness (Lawton, Gramatki, Watt, & Fujiwara, 2020), reduced depression and better well‐being (Jenkinson et al., 2013; Thoits & Hewitt, 2001), as well as being valuable for vulnerable groups, such as refugees (Carlton, 2015). Prosocial behaviour also offers benefits that are known to reduce depression. For example, helping others encourages perspective‐taking and reappraisal (Davis & Maitner, 2010), provides social approval via the enactment of social norms (Oarga et al., 2015), increases self‐esteem and efficacy (Ellemers & Boezeman, 2010) and improves emotion regulation (Doré, Morris, Burr, Picard, & Ochsner, 2017).

Prosocial behaviour has also been shown to reduce loneliness (Joloza, 2013) and is linked with the building of health‐enhancing social capital and trust (Pilkington, Windsor, & Crisp, 2012; Poortinga, 2006): collective resources capable of reducing threat and uncertainty (Siegrist, Gutscher, & Earle, 2005). Moreover, it is these social aspects that appear to influence the relationships between helping behaviours and depression (Creaven, Healy, & Howard, 2018), through the generation of a sense of meaning (Thoits, 2012), of “mattering” to others (Piliavin, 2010), and social connectedness (Musick & Wilson, 2003). This is especially true for those who lacked social integration before volunteering (Piliavin, 2010).

Shared social identification is a well‐known predictor of help‐giving (e.g., Levine, Prosser, Evans, & Reicher, 2005). However, beneficial social connectedness can also be driven by help‐giving. Indeed, ingroup helping and the solidarity it fosters are considered crucial social bonding mechanisms (Reicher & Haslam, 2010), and research has demonstrated that volunteering can enhance a sense of community (Casiday, Kinsman, Fisher, & Bambra, 2008; Omoto, Snyder, & Hackett, 2010). From this perspective, community belonging and its “social cure” resources may be generated by help‐giving. Indeed, interviews with volunteers revealed that their work resulted in an enhanced sense of community and belonging (Gray & Stevenson, 2020). Moreover, the strong relationships with the community built by volunteering also predicts increased personal well‐being through community identification and elevated perceptions of social support (Bowe et al., 2020). In other words, volunteering serves to build community identification, thereby unlocking enhanced “social cure” effects.

### 1.4 The present study

Taken together, prior research indicates that cohesive communities positively impact upon the health of residents and, in times of crisis, form the basis for solidarity, unity and coordinated collective action. Helping behaviour does not merely arise from community belonging but plays an important role in building community identification and togetherness thus influencing mental health and well‐being through the resultant sharing of social and psychological resources (in addition to the direct benefits accrued by the help recipients). In terms of responding to the challenges of COVID‐19, we expect this theoretical framework to reveal the pathways through which participation in the globally observed community aid phenomena may predict the mental health and well‐being of residents engaged in those crucial community support behaviours. Specifically, we predict that taking part in organized helping in the context of COVID‐19 will predict better well‐being (H1) and reduced levels of the negative mental health outcomes most associated with COVID‐19's social threats: depression and anxiety (H2). Finally, as well‐being has been found to be influenced by community belonging and togetherness during disaster, we expect these relationships will be mediated by increases in community identification and feelings of unity in response to the pandemic (H3).

## 2 METHOD

### 2.1 Participants and procedure

Two‐hundred‐and‐five adults living in England (60 males, 145 females; *M*_age_ = 35.44 years, *SD* = 11.44, *range* = 18–72) were recruited through prolific academic and paid £3.75 to complete an online survey on June 1, 2020. The survey contained several modules exploring various psychological phenomena. Sample size was deemed suitable for the analysis of these variables.^1^ Means of items were obtained for scales.

### 2.2 Measures

C*oordinated community help during the COVID‐19 pandemic* was measured with an adaptation of the Provided and Received Coordinated Social Support Scale (Drury et al., 2016).^2^ Participants were asked the following: “Since the start of the coronavirus pandemic, to what extent have you done each of the following in response to the coronavirus pandemic?”, and then rated their agreement with six items that asked about their engagement in COVID‐19‐related helping (e.g., “I have participated in one or more groups that were created in order to support members of my local community”) on a 1 (“Not at all”) to 5 (”To a very great extent”) scale. Higher values indicated more participation in coordinated helping since the start of the pandemic. Reliability was good (α = .82).

*Community identification* was measured with a single‐item Group Identification Measure (Postmes, Haslam, & Jans, 2013), “I identify as a member of my local community”, using a 1 (“I strongly disagree”) to 7 (“I strongly agree”) scale.

S*ense of unity during the COVID‐19 pandemic* (hereafter *unity*) was measured with an adaptation of the Sense of Unity After Disaster scale (Kaniasty, 2012). Participants rated their agreement with each of the four items (e.g., “Since the start of the coronavirus pandemic, I have felt as part of a united community of people who experienced a shared misfortune”) using a 1 (”I strongly disagree”) to 7 (“I strongly agree”) scale. Although Cronbach' alpha was relatively low (α = .61), removing any item reduced the value, so all items were retained.

*Well‐being* was measured with the four‐item Personal Well‐being Score (Benson, Sladen, Liles, & Potts, 2019). Participants rated their agreement with each item (e.g., “Overall, how satisfied are you with your life nowadays?”) on a 0 (“Not at all”) to 10 (“Completely”) scale. Higher values indicated better well‐being. Reliability was good (α = .80).

*Depression* was measured with the nine‐item Patient Health Questionnaire (PHQ‐9) scale (Kroenke & Spitzer, 2002). Participants rated the frequency of symptoms experienced over the past 2 weeks (e.g., “Little interest or pleasure in doing things”) on a 1 (“Not at all”) to 4 (“Nearly every day”) scale. Items were summed, with higher values indicating higher depression (α = .89).

A*nxiety* was measured with the seven‐item General Anxiety Disorder (GAD‐7) scale (Spitzer, Kroenke, Williams, & Löwe, 2006). Participants rated the frequency of symptoms experienced over the past 2 weeks (e.g., “Feeling nervous, anxious or on edge”) on a 1 (“Not at all”) to 4 (“Nearly every day”) scale. Items were summed, with higher values indicating higher anxiety (α = .91).

*Demographic information* was then gathered, including age, gender and whether the participant was in a romantic relationship.

## 3 RESULTS

### 3.1 Descriptive statistics and correlations

Descriptive statistics and partial correlations (controlling for age, gender and relationship status) are provided in Table 1. Coordinated Community Helping During COVID‐19 Scale scores reveal that whilst few people selected “to a very great extent” to indicate the extent of their participation in pandemic‐related coordinated helping (between 1% and 4.9% of respondents selected this option across the six items; mean = 2.83%), greater numbers of participants were engaged in lower level coordinated helping (between 6.3% and 26.3% selected “to a small extent” across all six items; mean = 17.38%). Scores across the items on the scale also revealed that more people in our sample tended to help by working together with neighbours than leading or organizing groups themselves.^3^ As expected, correlational analyses revealed that coordinated help‐giving correlated positively with community identification, unity and well‐being. Community identification also correlated positively with unity, and well‐being, and negatively with depression and anxiety, while unity correlated positively with well‐being and negatively with depression and anxiety.

**TABLE 1:** Descriptive statistics and partial correlations (Controlling for age, gender and relationship status)

	1	2	3	4	5	6
1.Giving coordinated help	—					
2.Community identification	.35***	—				
3.Unity during pandemic	.18**	.24**	—			
4.Well‐being	.16**	.18**	.34***	—		
5.Depression	−.05	−.17*	−.29***	−.68***	—	
6.Anxiety	−.02	−.15*	−.37***	−.64***	.76***	—
*M*	1.73	4.63	4.49	6.25	17.20	14.27
*SD*	0.81	1.47	1.02	1.97	6.03	5.22

*Note*: ***p ≤ .001, **p ≤ .01, *p < .05. Variable response ranges: Giving coordinated help: 1–5, community identification: 1–7, unity during pandemic: 1–7, well‐being: 0–10, depression: 9–36, anxiety: 7–28. At the bottom of each column, the first value is the variable's mean and the second value is the variable's *SD*.

### 3.2 Indirect effects analyses

Three indirect effects analyses were conducted to explore the extent to which coordinated help‐giving predicted well‐being/mental health through two serial mediators representing community identity and connectedness during the pandemic: community identification and unity. Model six in version 3.4 of Hayes' (2017) PROCESS macro was used, with the analyses involving 10,000 bootstrapping samples with 95% confidence intervals (LLCI/ULCI), using the percentile method.^4^ Gender, age and relationship status were controlled for. See Table 2 for a numerical summary of each model. For completeness, all analyses were re‐run with the positions of the mediators reversed (i.e., unity before community identification). These alternative models were non‐significant, indicating that the hypothesized ordering of mediators produced stronger models.

**TABLE 2:** Summary of the serial mediation analyses

Giving coordinated help predicts well‐being via community identification and Unity	
Indirect effect	*Effect* = .05, *Boot SE* = .03, *Boot LLCI* = .007, *Boot ULCI* = .13
Path from coordinated help‐giving to community identification	*b* = .62, *SE* = .12, *t* = 5.22, *p* < .001, *LLCI* = .39, *ULCI* = .86
Path from community identification to Unity during pandemic	*b* = .14, *SE* = .05, *t* = 2.71, *p* = .007, *LLCI* = .04, *ULCI* = .24
Path from unity during pandemic to well‐being	*b* = .59, *SE* = .13, *t* = 4.47, *p* < .001, *LLCI* = .33, *ULCI* = .85
Total effect	*Effect* = .39, *SE* = .17, *t* = 2.27, *p* = .02, *LLCI* = .05, *ULCI* = .72
Direct effect	*Effect* = .18, *SE* = .17, *t* = 1.03, *p* = .30, *LLCI* = −.16, *ULCI* = .52.

Giving coordinated help predicts depression via community identification and unity	
Indirect effect	*Effect* = −.14, *Boot SE* = .09, *Boot LLCI* = −.36, *Boot ULCI* = −.01
Path from coordinated help‐giving to community identification	*b* = .62, *SE* = .12, *t* = 5.22, *p* < .001, *LLCI* = .39, *ULCI* = .86
Path from community identification to unity during pandemic	*b* = .14, *SE* = .05, *t* = 2.71, *p* = .007, *LLCI* = .04, *ULCI* = .24
Path from unity during pandemic to depression	*b* = −1.57, *SE* = .41, *t* = −3.84, *p* = .0002, *LLCI* = −2.38, *ULCI* = −.76
Total effect	*Effect* = −.38, *SE* = .52, *t* = −.74, *p* = .46, *LLCI* = −1.41, *ULCI* = .64
Direct effect	*Effect* = .29, *SE* = .53, *t* = .54, *p* = .59, *LLCI* = −.76, *ULCI* = 1.34

Giving coordinated help predicts anxiety via community identification and unity	
Indirect effect	*Effect* = −.16, *Boot SE* = .10, *Boot LLCI* = −.42, *Boot ULCI* = −.02
Path from coordinated help‐giving to community identification	*b* = .62, *SE* = .12, *t* = 5.22, *p* < .001, *LLCI* = .39, *ULCI* = .86
Path from community identification to unity during pandemic	*b* = .14, *SE* = .05, *t* = 2.71, *p* = .007, *LLCI* = .04, *ULCI* = .24
Path from unity during pandemic to anxiety	*b* = −1.84, *SE* = .34, *t* = −5.36, *p* < .001, *LLCI* = −2.51, *ULCI* = −1.16
Total effect	*Effect* = −.13, *SE* = .45, *t* = −.29, *p* = .77, *LLCI* = −1.02, *ULCI* = .76
Direct effect	*Effect* = .51, *SE* = .45, *t* = 1.13, *p* = .26, *LLCI* = −.38, *ULCI* = 1.39

### 3.3 Giving coordinated help predicts well‐being via community identification and unity

**FIGURE 1:**
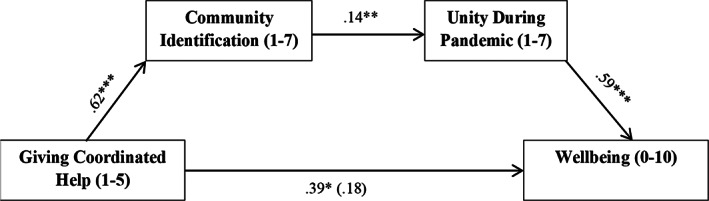
Model depicting the significant indirect effect of giving coordinated help on well‐being via community identification and sense of unity during the pandemic. Gender, age and relationship status were controlled for in the analysis, but are not shown. Bracketed coefficient is the direct effect. *Note*: ***p < .001, ** *p* < .01, *p < .05

As predicted, there was a significant indirect effect of giving of coordinated help on well‐being though community identification and unity. Coordinated help‐giving was a positive predictor of community identification, while community identification was a positive predictor of unity, and unity was a positive predictor of well‐being. The total effect of coordinated help‐giving on well‐being was significant and became non‐significant when both mediators were accounted for (i.e., the direct effect), indicating full mediation (see Figure 1).

### 3.4 Giving coordinated help predicts depression via community identification and unity

**FIGURE 2:**
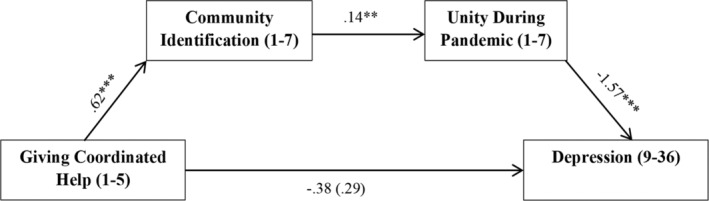
Model depicting the significant indirect effect of giving coordinated help on depression via community identification and sense of unity during the pandemic. Gender, age and relationship status were controlled for in the analysis, but are not shown. Bracketed coefficient is the direct effect. *Note*: ***p < .001, ** *p* < .01, *p < .05

As predicted, there was a significant indirect effect of coordinated help‐giving on depression though community identification and unity. Coordinated help‐giving was a positive predictor of community identification, whilst community identification was a positive predictor of unity, and unity was a negative predictor of depression. The total effect of coordinated help‐giving on depression was non‐significant, and this remained non‐significant when the mediators were accounted for (i.e., the direct effect; see Figure 2).^5^

### 3.5 Giving coordinated help predicts anxiety via community identification and unity

**FIGURE 3:**
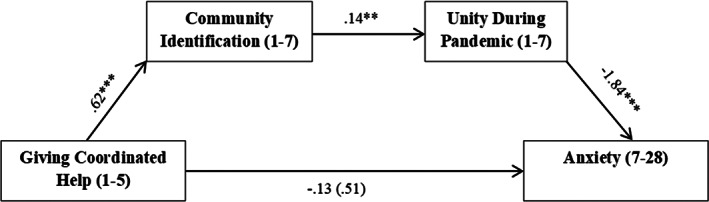
Model depicting the significant indirect effect of giving coordinated help on anxiety via community identification and sense of unity during the pandemic. Gender, age and relationship status were controlled for in the analysis, but are not shown. Bracketed coefficient is the direct effect. *Note*: ***p < .001, ** *p* < .01, *p < .05

As predicted, a significant indirect effect of coordinated help‐giving on anxiety through community identification and unity was found. Coordinated help‐giving was a positive predictor of community identification, whilst community identification was a positive predictor of unity, and unity was a negative predictor of anxiety. The total effect of coordinated help‐giving on anxiety was non‐significant, and this remained non‐significant when the mediators were accounted for (i.e., the direct effect; see Figure 3).

## 4 DISCUSSION

Despite dominant media narratives of panic and self‐centred behaviour, historical analyses reveal humans' great capacity to overcome emergencies with positive, coordinated and altruistic action (e.g., Solnit, 2010). These responses, and those exhibited in response to the COVID‐19 pandemic, cannot be explained with reference to the sum of individual actions and tendencies but instead must be explained in social psychological terms at the group level (Ntontis & Rocha, 2020). The local community is one such group. Community responses have a critical role to play during times of crisis, reinforcing a sense of common fate, shared identity and community support (Ntontis et al., 2018). During times of challenge, these unifying group experiences also bring about a valuable resource in the form of prosocial behaviours (Alfadhli et al., 2019; Vezzali et al., 2016). This was especially evident during the COVID‐19 pandemic's early stages, when there was an upsurge in the delivery of mutual aid via community‐based volunteering (Drury & Tekin Guven, 2020; Monbiot, 2020). Whilst the benefits of this response are clear for recipients, the current study shifts focus to illustrate potential health benefits for volunteers themselves.

In doing so, the findings contribute to a growing body of work demonstrating the utility of the Social Cure approach (C. Haslam et al., 2018) for identifying specific pathways through which community‐based helping benefits volunteers' well‐being (Bowe et al., 2020; Gray & Stevenson, 2020). Thus, although correlational, the current data corroborate other mixed methods work in this relatively understudied area of the social cure tradition (Bowe et al., 2020) showing how specific behaviours not only result from shared social relationships but can also actively contribute to the maintenance and building of social identities. Going further, the present findings further extend this literature by synthesizing insights from the social psychology of disaster responses (e.g., Drury et al., 2016) to show how these pathways between prosocial behaviour, community relationships and well‐being operate during the current global health crisis and by revealing the role of community unity. Whilst these relationships have, to date, been extensively theorized, our work is the first (to our knowledge) to demonstrate how community help‐giving during the pandemic predicts better mental health via increased social identification.

As discussed, the pandemic poses threats to global social cohesion (Banerjee & Rai, 2020; O'Connor et al., 2020): a feature known to be fundamental to health (C. Haslam et al., 2018). It is thus vital to consider how to alleviate pandemic‐related social disconnection. During disasters, unifying community responses can buffer against distress and even boost mental well‐being (Drury et al., 2009, 2020), and this is partially linked to solidarity and help‐giving (Vezzali et al., 2016). However, recent literature exploring such strategies focusses primarily on community identities that emerge in response to emergencies or disasters (e.g., Ntontis et al., 2018). This literature has effectively demonstrated how shared experiences and common fate can lead to solidarity and coordinated community responses. Whilst our research does not explore the potential impact of common fate on identification and helping, and thus cannot speak to these processes, we show instead how communities may bond further by actively helping and supporting vulnerable residents. In doing so, we demonstrate that these behaviours are not only closely linked with strength of community identification and a sense of unity during these stressful events, but they also predict the mental health and well‐being of the help‐givers. Furthermore, whilst previous research has illustrated that community identification enhances residents' well‐being through psychological resource provision (e.g., collective efficacy; McNamara et al., 2013), the current study suggests a novel pathway through sense of unity during the pandemic. This unity is considered essential, given the physical distancing and restricted movement required to manage the pandemic and the risks associated with physical isolation (Elcheroth & Drury, 2020). Our results suggest that, for those able to participate, community‐based helping may help overcome at least some of the pandemic‐associated threats of social disconnection. However, further work is needed to determine the causal processes within these relationships.

As well as offering a route to unity and community belongingness, our study responds to the need to support community members' health/well‐being using scientific investigation (Holmes et al., 2020; Van Bavel et al., 2020). Acknowledging concerns about the strain placed on volunteers/voluntary services as they bolster governmental responses (Gilbert, 2020), we explored whether the well‐established positive outcomes of volunteering persisted during these extraordinary times. Our study demonstrates that, at least in the short‐term, there are benefits associated with community help‐giving, supporting previous work on the benefits of informal help‐giving (Thoits & Hewitt, 2001). As well as demonstrating strong connections between well‐being and help‐giving, our findings are promising in terms of suggesting that help‐giving may be a way to reduce pandemic‐related mental distress (Lee et al., 2020; Wang et al., 2020).

The findings of this study suggest that supporting community members to engage in community‐based volunteering and mutual aid that allows them to connect with other community members is vital for multiple reasons. Given the value and situated nature of coordinated community helping resources, and the observed relationships between participation, connection and health/well‐being, targeted support to enable and resource such community initiatives to function cohesively over the longer term represents a valuable investment. Existing research on health initiatives such as Social Prescribing have already evidenced the value inherent in investments in community‐based participation for individuals experiencing the health‐related effects of social isolation (Kellezi et al., 2019; Wakefield et al., 2020). These findings contribute to this growing body of literature revealing the psychological assets embedded within local social groups: assets which have become even more important during this period of unanticipated distress and which can be observed in the form of unifying community helping responses that benefit both the helped and helper.

## CONFLICT OF INTEREST

The authors declare no conflicts of interest.

## Supporting information


**Data S1.** Supporting information.

## Data Availability

The materials and data supporting this study have been archived on a secure server at Nottingham Trent University. Access to the data used in this article is available on The Open Science Framework at https://osf.io/vtseb/?view_only=bc1eca351a1645b39ce38f03be6c188b.

## Endnotes

^1^
An a priori sample size of 103 was calculated for a mediation model containing seven variables (one predictor, two mediators, one outcome, three control variables), assuming 0.80 power and a medium‐sized *f* ^2^ of 0.15.

^2^
Adapted measures are provided in full in Data S1.

^3^
Distribution of scores across all items of this measure are provided in Data S1.

^4^
The bootstrapping re‐sampling procedure involves no assumptions regarding the distribution of the data (Preacher et al., 2007), making it particularly suitable for use with small or skewed samples.

^5^
Note that it is still appropriate to explore indirect effects models when the total effect is non‐significant (this is known as indirect‐only mediation; Zhao, Lynch Jr, & Chen, 2010).
