# Supplementary material for: The mental health benefits of community helping during crisis: Coordinated helping, community identification and sense of unity during the COVID‐19 pandemic
Source: J Community Appl Soc Psychol. 2021 Apr 5;32(3):521–35. doi: 10.1002/casp.2520 (PMC8250747; doi:10.1002/casp.2520)
Supplement: Supplementary file 1 — Data S1. Supporting information. [file CASP-32-521-s001.docx]

**Supplementary Materials**

Table 3

Distribution of scores on the Coordinated Community Helping during the COVID-19 Pandemic Scale (percentage of total participants selecting each item response option in parenthesis)

|  | Not at all | To a small extent | To a moderate extent | To a great extent | To a very great extent | Mean Score (SD) |
| --- | --- | --- | --- | --- | --- | --- |
|  |  |  |  |  |  |  |
| *I have participated in one or more groups that were created in order to support members of my local community* | 106 (51.7) | 42 (20.5) | 35 (17.1) | 16 (7.8) | 6 (2.9) | 1.90 (1.12) |
| *I have worked together with neighbours I already knew* | 81 (39.5) | 54 (26.3) | 44 (21.5) | 20 (9.8) | 6 (2.9) | 2.10 (1.12) |
| *I have worked with previously unknown neighbours* | 126 (61.5) | 41 (20) | 17 (8.3) | 15 (7.3) | 6 (2.9) | 1.70 (1.08) |
| *I have participated in helping members of vulnerable groups at risk of Coronavirus within my community* | 104 (50.7) | 42 (20.5) | 34 (16.6) | 15 (7.3) | 10 (4.9) | 1.95 (1.19) |
| *I have participated in helping people infected by Coronavirus within my community* | 154 (75.1) | 22 (10.7) | 16 (7.8) | 8 (3.9) | 5 (2.4) | 1.48 (.97) |
| *I have organised or led a group/team/organisation intended to support members of my local community* | 178 (86.8) | 13 (6.3) | 9 (4.4) | 3 (1.5) | 2 (1) | 1.23 (.60) |
|  |  |  |  |  |  |  |

Note: *N*=205 participants.

Table 4

Adapted Scale Items: Giving of coordinated community help during the COVID-19 pandemic

| Item 1  Item 2  Item 3  Item 4  Item 5  Item 6 | I have participated in one or more groups that were created in order to support members of my local community  I have worked together with neighbours I already knew  I have worked with previously unknown neighbours  I have participated in helping members of vulnerable groups at risk of Coronavirus within my community  I have participated in helping people infected by Coronavirus within my community  I have organised or led a group/team/organisation intended to support members of my local community |
| --- | --- |

Table 5

Adapted Scale Items: Sense of unity during the COVID-19 pandemic

| Item 1  Item 2  Item 3  Item 4 | Since the start of the Coronavirus pandemic, people have united and prior disagreements and differences among people disappeared  Since the start of the Coronavirus pandemic, I have felt rejected and neglected by other people *(reverse scored)*  Since the start of the Coronavirus pandemic, people have been selfish and have cared only for themselves *(reverse scored)*  Since the start of the Coronavirus pandemic, I have felt as part of a united community of people who experienced a shared misfortune |
| --- | --- |
